# A new mixed-valence lead(II) mangan­ese(II/III) phosphate(V): PbMn^II^_2_Mn^III^(PO_4_)_3_

**DOI:** 10.1107/S1600536813016504

**Published:** 2013-06-22

**Authors:** Ghaleb Alhakmi, Abderrazzak Assani, Mohamed Saadi, Lahcen El Ammari

**Affiliations:** a Laboratoire de Chimie du Solide Appliquée, Faculté des Sciences, Université Mohammed V-Agdal, Avenue Ibn Battouta, BP 1014, Rabat, Morocco

## Abstract

The title compound, lead trimanganese tris(orthophosphate), has been synthesized by hydro­thermal methods. In this structure, only two O atoms are in general positions and all others atoms are in the special positions of the *Imma* space group. Indeed, the atoms in the Wyckoff positions are namely: Pb1 and P1 on 4*e* (*mm*2); Mn1 on 4*b* (2/*m*); Mn2 and P2 on 8*g* (2); O1 on 8*h* (*m*); O2 on 8*i* (*m*). The crystal structure can be viewed as a three-dimensional network of corner- and edge-sharing PO_4_ tetra­hedra and MnO_6_ octa­hedra, building two types of chains running along the *b* axis. The first is an infinite linear chain, formed by alternating Mn^III^O_6_ octa­hedra and PO_4_ tetra­hedra which share one vertex. The second chain is built up from two adjacent edge-sharing octa­hedra (Mn^II^_2_O_10_ dimers) whose ends are linked to two PO_4_ tetra­hedra by a common edge. These chains are linked together by common vertices of polyhedra in such a way as to form porous layers parallel to (001). These sheets are bonded by the first linear chains, leading to the appearance of two types of tunnels, one propagating along the *a* axis and the other along *b*. The Pb^II^ ions are located within the inter­sections of the tunnels with eight neighbouring O atoms in form of a trigonal prism that is capped by two O atoms on one side. The three-dimensional framework of this structure is compared with similar phosphates such as Ag_2_Co_3_(HPO_4_)(PO_4_)_2_ and Ag_2_Ni_3_(HPO_4_)(PO_4_)_2_.

## Related literature

For compounds with related structures see: Assani *et al.* (2011*a* ▶,*b* ▶,*c* ▶); Moore & Ito (1979 ▶). For applications of related compounds, see: Trad *et al.* (2010 ▶). For compounds with mixed-valence manganese(II/III) lead(II) triphosphates(V), see: Adam *et al.* (2009 ▶). For bond-valence analysis, see: Brown & Altermatt (1985 ▶).

## Experimental

### 

#### Crystal data

PbMn_3_(PO_4_)_3_
*M* *_r_* = 656.92
Orthorhombic, 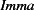

*a* = 10.2327 (8) Å
*b* = 13.9389 (9) Å
*c* = 6.6567 (4) Å
*V* = 949.46 (11) Å^3^
*Z* = 4
Mo *K*α radiation
μ = 22.15 mm^−1^
*T* = 296 K
0.36 × 0.23 × 0.10 mm

#### Data collection

Bruker X8 APEX diffractometer
Absorption correction: multi-scan (*SADABS*; Bruker, 2009 ▶) *T*_min_ = 0.046, *T*_max_ = 0.215
4704 measured reflections
787 independent reflections
771 reflections with *I* > 2σ(*I*)
*R*_int_ = 0.028

#### Refinement

*R*[*F* ^2^ > 2σ(*F* ^2^)] = 0.016
*wR*(*F* ^2^) = 0.040
*S* = 1.11
787 reflections
53 parameters
Δρ_max_ = 2.54 e Å^−3^
Δρ_min_ = −0.98 e Å^−3^

### 

Data collection: *APEX2* (Bruker, 2009 ▶); cell refinement: *SAINT* (Bruker, 2009 ▶); data reduction: *SAINT*; program(s) used to solve structure: *SHELXS97* (Sheldrick, 2008 ▶); program(s) used to refine structure: *SHELXL97* (Sheldrick, 2008 ▶); molecular graphics: *ORTEP-3 for Windows* (Farrugia, 2012 ▶) and *DIAMOND* (Brandenburg, 2006 ▶); software used to prepare material for publication: *publCIF* (Westrip, 2010 ▶).

## Supplementary Material

Crystal structure: contains datablock(s) I, global. DOI: 10.1107/S1600536813016504/br2227sup1.cif

Structure factors: contains datablock(s) I. DOI: 10.1107/S1600536813016504/br2227Isup2.hkl

Additional supplementary materials:  crystallographic information; 3D view; checkCIF report

## Figures and Tables

**Fig. 1.**
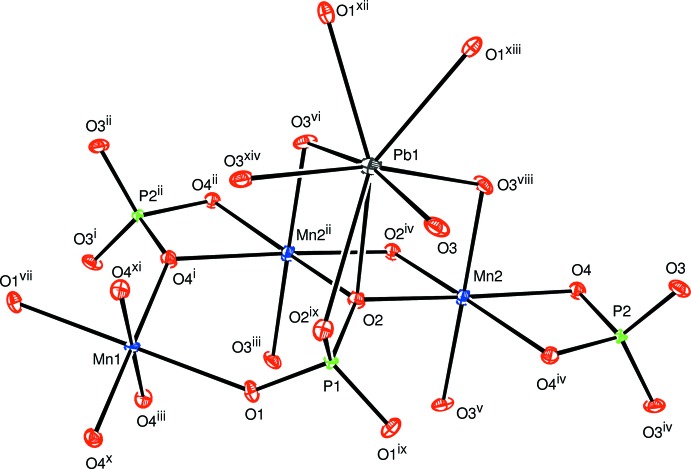
A partial three-dimensional plot of the crystal structure of the title compound. Displacement ellipsoids are drawn at the 50% probability level. Symmetry codes:(i) -x, y + 1/2, -z; (ii) -x, -y, -z; (iii) x, -y + 1/2, z; (iv) -x, -y, -z + 1; (v) -x - 1, -y, -z; (vi) x + 1, y, z; (vii) -x, -y + 1, -z + 1; (viii) x + 1, -y + 1/2, z + 1; (ix) x + 1, y + 1, z; (x) x, -y - 1/2, z; (xi) x - 1, y - 1, z; (xii) -x, y - 1/2, -z + 1; (xiii) -x - 1, y + 1/2, -z;

**Fig. 2.**
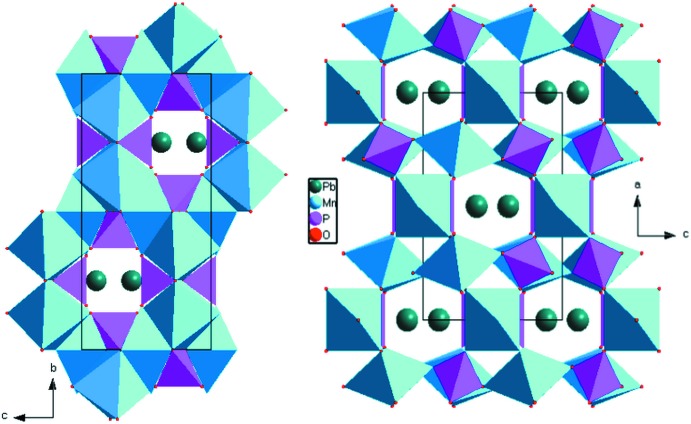
Polyhedral representation of PbMn3(PO4)3 showing tunnels running along the a and b directions.

## supplementary crystallographic information

### Comment

In a previous hydrothermal investigation of the system A_2_O—MO—P_2_O_5_, we have succeeded to synthesize and structurally characterize new phosphate like Ag_2_*M*_3_(HPO_4_)(PO_4_)_2_ (*M* = Co, Ni) which crystallizes in orthorhombic system with Ima2 space group (Assani *et al.*, 2011*a*, 2011*b*). The structure of this compound is an open frameworks such as that observed in the alluaudite. On the basis of crystallographic considerations, the later phosphates structure represents similarities with the well known alluaudite structure. Accordingly, both structures can be represented by the general formula A(1) A(2)*M*(1)*M*(2)_2_(PO_4_)_3_ as it has been established by Moore & Ito, 1979, in the case of alluaudite related compounds. This model is written according to decreasing size of the discrete sites. Mainly, the A sites can be occupied by either mono- or divalent medium-sized cations while the *M* cationic site corresponds to an octahedral environment generally occupied by the transition metal cations. This fact, particularly in the case of the alluaudite structure, has offered a great field of application as positive electrode in the lithium and sodium batteries (Trad *et al.*, 2010).

In accordance with the forefront of our research, a focus of investigation is associated with the mixed cation orthophosphates belonging to the above-mentioned compounds. Hence, by means of the hydrothermal method, we have recently synthesized and structurally characterized numerous phosphates corresponding to the general formula A(1) A(2)*M*(1)_2_M(2)(PO_4_)_3_ (Assani *et al.*, 2011*c*). The present paper is devoted to one of them, namely, PbMn^2+^_2_Mn^3+^(PO_4_)_3_, with manganese mixed valence, rarely encountered in the literature (Adam *et al.*, 2009).

A partial three-dimensional plot of the crystal structure of the title compound is represented in Fig. 1, illustrating the connection of the metal-oxygen polyhedra. All atoms of this structure are in special positions, except two oxygen atoms (O3,O4) in general position of the Imma space group. The crystal structure network consists of single phosphate PO_4_ tetrahedron linked to MnO_6_ octahedra, building two types of chains running along the *b* axis. The first chain is formed by an alternating Mn^III^O_6_ octahedron and PO_4_ tetrahedron which share one vertex. The second chain is built up from two adjacent edge sharing octahedra (Mn^II^_2_O_10_, dimers) whose ends are linked to two PO_4_ tetrahedra by a common edge. These two types of chains are linked together by common vertex of plyhedra to form porous sheets parallel to the (0 0 1) plane. The three dimensional framework delimits two types of tunnels parallel to a and b directions where the lead atoms are located as shown in Fig.2. Each lead cation is surrounded by 8 oxygenes.

Bond valence sum calculationlead(II) manganese(II/III) triphosphate(V): PbMn_3_(PO_4_)_3_s (Brown & Altermatt, 1985) for Pb1^2+^, Mn1^3+^, Mn2^2+^, P1^5+^ and P2^5+^ ions are as expected, *viz*. 1.796, 3.042, 1.975, 5.014 and 4.843 valence units, respectively. The values of the bond valence sums calculated for all oxygen atoms are as expected in the range of 1.79 – 2.04 valence units. The three-dimensional framework of this structure is compared with some phosphates like Ag_2_*M*_3_(HPO_4_)(PO_4_)_2_ with *M* = Ni or Co, wherein the two Ag^+^ cations in the tunnels are replaced by Pb^2+^.

### Experimental

The crystal of the title compound is isolated from the hydrothermal treatment of the reaction mixture of lead, manganese and phosphate precursors in a proportion corresponding to the molar ratio Pb:Mn:*P* = 1: 3:3. The hydrothermal reaction was conducted in a 23 ml Teflon-lined autoclave, filled to 50% with distilled water and under autogeneous pressure at 483 K for five days. After being filtered off, washed with deionized water and air dried, the reaction product consists of a brown sheet shaped crystals corresponding to the title compound besides a light brown powder.

### Refinement

The highest peak and the deepest hole in the final Fourier map are at 0.78 Å and 0.76 Å, respectively, from Pb1. The not significant bonds and angles were removed from the CIF file.

### Crystal data

**Table:**

PbMn_3_(PO_4_)_3_	*F*(000) = 1192
*M**_r_* = 656.92	*D*_x_ = 4.596 Mg m^−^^3^
Orthorhombic, *I**m**m**a*	Mo *K*α radiation, λ = 0.71073 Å
Hall symbol: -I 2b 2	Cell parameters from 787 reflections
*a* = 10.2327 (8) Å	θ = 2.9–30.5°
*b* = 13.9389 (9) Å	µ = 22.15 mm^−^^1^
*c* = 6.6567 (4) Å	*T* = 296 K
*V* = 949.46 (11) Å^3^	Sheet, brown
*Z* = 4	0.36 × 0.23 × 0.10 mm

### Data collection

**Table:**

Bruker X8 APEX diffractometer	787 independent reflections
Radiation source: fine-focus sealed tube	771 reflections with *I* > 2σ(*I*)
Graphite monochromator	*R*_int_ = 0.028
φ and ω scans	θ_max_ = 30.5°, θ_min_ = 2.9°
Absorption correction: multi-scan (*SADABS*; Bruker, 2009)	*h* = −14→14
*T*_min_ = 0.046, *T*_max_ = 0.215	*k* = −19→19
4704 measured reflections	*l* = −9→9

### Refinement

**Table:**

Refinement on *F*^2^	0 restraints
Least-squares matrix: full	Primary atom site location: structure-invariant direct methods
*R*[*F*^2^ > 2σ(*F*^2^)] = 0.016	Secondary atom site location: difference Fourier map
*wR*(*F*^2^) = 0.040	*w* = 1/[σ^2^(*F*_o_^2^) + (0.0205*P*)^2^ + 2.635*P*] where *P* = (*F*_o_^2^ + 2*F*_c_^2^)/3
*S* = 1.11	(Δ/σ)_max_ < 0.001
787 reflections	Δρ_max_ = 2.54 e Å^−^^3^
53 parameters	Δρ_min_ = −0.98 e Å^−^^3^

### Special details

**Table:**

Geometry. All s.u.'s (except the s.u. in the dihedral angle between two l.s. planes) are estimated using the full covariance matrix. The cell s.u.'s are taken into account individually in the estimation of s.u.'s in distances, angles and torsion angles; correlations between s.u.'s in cell parameters are only used when they are defined by crystal symmetry. An approximate (isotropic) treatment of cell s.u.'s is used for estimating s.u.'s involving l.s. planes.
Refinement. Refinement of *F*^2^ against all reflections. The weighted *R*-factor *wR* and goodness of fit *S* are based on *F*^2^, conventional *R*-factors *R* are based on *F*, with *F* set to zero for negative *F*^2^. The threshold expression of *F*^2^ > 2σ(*F*^2^) is used only for calculating *R*-factors(gt) *etc*. and is not relevant to the choice of reflections for refinement. *R*-factors based on *F*^2^ are statistically about twice as large as those based on *F*, and *R*- factors based on all data will be even larger.

### Fractional atomic coordinates and isotropic or equivalent isotropic displacement parameters (Å^2^)

**Table:**

	*x*	*y*	*z*	*U*_iso_*/*U*_eq_	
Pb1	0.0000	0.2500	−0.11391 (3)	0.01308 (7)	
Mn1	0.0000	0.0000	0.5000	0.00488 (14)	
Mn2	0.2500	0.36754 (4)	0.2500	0.00774 (12)	
P1	0.0000	0.2500	0.40554 (17)	0.0044 (2)	
P2	0.2500	0.57316 (6)	0.2500	0.00507 (17)	
O1	0.0000	0.16010 (18)	0.5362 (4)	0.0086 (5)	
O2	0.1182 (3)	0.2500	0.2619 (4)	0.0084 (5)	
O3	0.2066 (2)	0.63321 (13)	0.0719 (3)	0.0101 (4)	
O4	0.36271 (18)	0.50018 (12)	0.1977 (3)	0.0078 (3)	

### Atomic displacement parameters (Å^2^)

**Table:**

	*U*^11^	*U*^22^	*U*^33^	*U*^12^	*U*^13^	*U*^23^
Pb1	0.01879 (13)	0.01275 (10)	0.00770 (10)	0.000	0.000	0.000
Mn1	0.0038 (3)	0.0069 (3)	0.0040 (3)	0.000	0.000	0.0000 (2)
Mn2	0.0093 (3)	0.0054 (2)	0.0085 (2)	0.000	−0.00019 (19)	0.000
P1	0.0044 (6)	0.0045 (5)	0.0044 (5)	0.000	0.000	0.000
P2	0.0056 (4)	0.0047 (3)	0.0049 (4)	0.000	0.0007 (3)	0.000
O1	0.0092 (13)	0.0067 (10)	0.0100 (11)	0.000	0.000	0.0033 (9)
O2	0.0069 (12)	0.0086 (10)	0.0098 (11)	0.000	0.0036 (9)	0.000
O3	0.0123 (10)	0.0118 (8)	0.0061 (7)	0.0036 (7)	0.0014 (7)	0.0030 (6)
O4	0.0070 (9)	0.0078 (7)	0.0086 (8)	−0.0002 (6)	0.0023 (7)	0.0005 (6)

### Geometric parameters (Å, º)

**Table:**

Pb1—O1^i^	2.645 (3)	Mn2—O3^iv^	2.1881 (18)
Pb1—O1^ii^	2.645 (3)	Mn2—O3^xii^	2.1881 (18)
Pb1—O3^iii^	2.683 (2)	Mn2—O4^xiii^	2.2067 (18)
Pb1—O3^iv^	2.683 (2)	Mn2—O4	2.2067 (18)
Pb1—O3^v^	2.683 (2)	Mn2—P2	2.8660 (10)
Pb1—O3^vi^	2.683 (2)	P1—O2^xiv^	1.542 (3)
Mn1—O4^vii^	1.9249 (18)	P1—O2	1.542 (3)
Mn1—O4^viii^	1.9249 (18)	P1—O1^xiv^	1.525 (3)
Mn1—O4^ix^	1.9249 (18)	P1—O1	1.525 (3)
Mn1—O4^x^	1.9249 (18)	P2—O3^xiii^	1.5180 (19)
Mn1—O1	2.245 (3)	P2—O3	1.5180 (19)
Mn1—O1^xi^	2.245 (3)	P2—O4^xiii^	1.5767 (19)
Mn2—O2	2.1237 (18)	P2—O4	1.5767 (19)
Mn2—O2^x^	2.1237 (18)		
			
O1^i^—Pb1—O1^ii^	56.57 (11)	O1—Mn1—O1^xi^	180.0
O1^i^—Pb1—O3^iii^	78.72 (5)	O2—Mn2—O2^x^	79.02 (12)
O1^ii^—Pb1—O3^iii^	112.30 (5)	O2—Mn2—O3^iv^	84.48 (8)
O1^i^—Pb1—O3^iv^	112.30 (5)	O2^x^—Mn2—O3^iv^	95.10 (9)
O1^ii^—Pb1—O3^iv^	78.72 (5)	O2—Mn2—O3^xii^	95.10 (9)
O3^iii^—Pb1—O3^iv^	168.02 (8)	O2^x^—Mn2—O3^xii^	84.48 (8)
O1^i^—Pb1—O3^v^	112.30 (5)	O3^iv^—Mn2—O3^xii^	179.45 (10)
O1^ii^—Pb1—O3^v^	78.72 (5)	O2—Mn2—O4^xiii^	107.97 (8)
O3^iii^—Pb1—O3^v^	74.73 (9)	O2^x^—Mn2—O4^xiii^	169.80 (8)
O3^iv^—Pb1—O3^v^	103.98 (9)	O3^iv^—Mn2—O4^xiii^	93.00 (7)
O1^i^—Pb1—O3^vi^	78.72 (5)	O3^xii^—Mn2—O4^xiii^	87.46 (7)
O1^ii^—Pb1—O3^vi^	112.30 (5)	O2—Mn2—O4	169.80 (8)
O3^iii^—Pb1—O3^vi^	103.98 (9)	O2^x^—Mn2—O4	107.97 (8)
O3^iv^—Pb1—O3^vi^	74.73 (9)	O3^iv^—Mn2—O4	87.46 (7)
O3^v^—Pb1—O3^vi^	168.02 (8)	O3^xii^—Mn2—O4	93.00 (7)
O4^vii^—Mn1—O4^viii^	180.0	O4^xiii^—Mn2—O4	66.18 (10)
O4^vii^—Mn1—O4^ix^	93.75 (11)	O2^xiv^—P1—O2	103.3 (2)
O4^viii^—Mn1—O4^ix^	86.25 (11)	O2^xiv^—P1—O1^xiv^	110.72 (7)
O4^vii^—Mn1—O4^x^	86.25 (11)	O2—P1—O1^xiv^	110.72 (7)
O4^viii^—Mn1—O4^x^	93.75 (11)	O2^xiv^—P1—O1	110.72 (7)
O4^ix^—Mn1—O4^x^	180.0	O2—P1—O1	110.72 (7)
O4^vii^—Mn1—O1	85.71 (7)	O1^xiv^—P1—O1	110.5 (2)
O4^viii^—Mn1—O1	94.29 (7)	O3^xiii^—P2—O3	113.08 (15)
O4^ix^—Mn1—O1	85.71 (7)	O3^xiii^—P2—O4^xiii^	113.41 (10)
O4^x^—Mn1—O1	94.29 (7)	O3—P2—O4^xiii^	108.31 (11)
O4^vii^—Mn1—O1^xi^	94.29 (7)	O3^xiii^—P2—O4	108.31 (11)
O4^viii^—Mn1—O1^xi^	85.71 (7)	O3—P2—O4	113.41 (10)
O4^ix^—Mn1—O1^xi^	94.29 (7)	O4^xiii^—P2—O4	99.65 (13)
O4^x^—Mn1—O1^xi^	85.71 (7)		

Symmetry codes: (i) *x*, *y*, *z*−1; (ii) −*x*, −*y*+1/2, *z*−1; (iii) −*x*, *y*−1/2, −*z*; (iv) *x*, −*y*+1, −*z*; (v) −*x*, −*y*+1, −*z*; (vi) *x*, *y*−1/2, −*z*; (vii) −*x*+1/2, *y*−1/2, *z*+1/2; (viii) *x*−1/2, −*y*+1/2, −*z*+1/2; (ix) *x*−1/2, *y*−1/2, *z*+1/2; (x) −*x*+1/2, −*y*+1/2, −*z*+1/2; (xi) −*x*, −*y*, −*z*+1; (xii) −*x*+1/2, −*y*+1, *z*+1/2; (xiii) −*x*+1/2, *y*, −*z*+1/2; (xiv) −*x*, −*y*+1/2, *z*.
